# Experimental Study on Multi-Directional Hybrid Energy Harvesting of a Two-Degree-of-Freedom Cantilever Beam

**DOI:** 10.3390/s25134033

**Published:** 2025-06-28

**Authors:** Minglei Han, Zhiqi Xing, Shuangbin Liu, Xu Yang

**Affiliations:** 1 School of Mechanical and Vehicle Engineering, Changchun University, Changchun 130022, China; 2 Key Laboratory of CNC Equipment Reliability, Ministry of Education, School of Mechanical and Aerospace Engineering, Jilin University, Changchun 130025, China

**Keywords:** hybrid energy harvesting, two-degree-of-freedom cantilever beam, multi-directional vibrations, electromagnetic energy harvester, piezoelectric energy harvester

## Abstract

Based on the research of the directional self-adaptive piezoelectric energy harvester (DSPEH), a structural design scheme of a multi-directional hybrid energy harvester (MHEH) is put forward. The working principle of the MHEH is experimentally studied. A prototype is designed and manufactured, and the output characteristics of the MHEH in vibrational degree of freedom (DOF) and rotational DOF are experimentally studied. Compared with the DSPEH, after adding the electromagnetic energy harvesting module, the MHEH effectively uses the rotational energy in the rotational DOF, achieves simultaneous energy harvesting from one excitation through two mechanisms, and the output power of the electromagnetic module reaches 61 μW. The total power of the system is increased by 10 times, the power density is increased by 500%, and the MHEH has high voltage output characteristics in multiple directions. Compared with traditional multi-directional and self-adaptive energy harvesters, the MHEH utilizes a reverse-thinking method to generate continuous rotational motion of the cantilever beam, thus eliminating the influence of external excitation direction on the normal vibration of the cantilever beam. In addition, the MHEH has achieved hybrid energy harvesting with a single cantilever beam and multiple mechanisms, providing new ideas for multi-directional energy harvesting.

## 1. Introduction

Vibration energy harvesting technology has made significant progress in recent years, especially driven by the rapid advancement of wireless sensor network (WSN) technology [1,2]. With the popularity of the Internet of Things (IoT) and intelligent monitoring systems, low-power sensor nodes are increasingly adopting self-powered technology to meet the long-term stable operation needs in complex environments such as industrial equipment monitoring, bridge health monitoring, aerospace, etc., while reducing dependence on traditional batteries and achieving the goal of a sustainable power supply [3,4,5].

Vibration energy harvesting technology converts mechanical vibration energy widely present in the environment into electrical energy through various electromechanical conversion mechanisms such as piezoelectric [6,7,8], electromagnetic [9,10,11], electrostatic [12], thermoelectric [13,14], and triboelectric methods [15,16,17]. Among them, piezoelectric energy harvesting technology has attracted much attention due to its simple structure and high energy density. A typical piezoelectric energy harvester adopts a cantilever beam structure and achieves electromechanical conversion through the piezoelectric effect of piezoelectric materials. However, environmental vibrations in practical applications often have directional randomness and frequency broadness, which leads to significant frequency matching difficulties and directional sensitivity in traditional single cantilever beam structures, severely restricting energy harvesting efficiency and application scope.

To overcome these limitations, researchers have proposed various innovative solutions. In terms of structural design, using multiple cantilever beams in series or parallel can broaden the frequency response range and achieve multi-directional energy harvesting [18,19,20,21,22,23,24]. For example, Zhou et al. [18] present a generalized theoretical model for predicting the structural dynamics and energy harvesting capability of FLZ energy harvesters. The experimental maximum output power of the energy harvester prototype at an excitation angle of 45° is 1.016 mW at an excitation level of 0.18 m s^−2^. Hung et al. [20] proposed a novel miniature three-axis vibration energy harvester with mechanical piezoelectric configuration. The output power of the harvester under x-axis vibration was 525.36 mV, 516.51 mV, and 548 mV. Su and Shih [22] propose a U-shaped multimodal bi-directional piezoelectric energy harvester, which can provide different resonant frequencies for different excitation directions.

The introduction of nonlinear magnetic coupling mechanisms and three-dimensional structural design can also effectively excite multi-directional vibrations [25,26,27,28,29,30,31,32,33,34,35,36,37,38]. For example, Fan et al. [27] propose a bidirectional hybrid energy harvester, which consists of two piezoelectric cantilever beams, a suspended magnet, and a set of coils. Two piezoelectric beams operate as the traditional piezoelectric energy harvester, while the suspended magnet and the coil form the two energy harvesting units of the electromagnetic energy harvester, achieving the simultaneous extraction of energy from one excitation through two conversion mechanisms. Wu et al. [31] propose an ultra-low-frequency multi-directional piezoelectric energy harvester based on a spring-loaded single pendulum system, which can efficiently harvest ultra-low-frequency vibration energy and can also harvest multi-directional vibration energy, and it can generate a high-power output of 13.29 mW at a lower operating frequency of 2.03 Hz.

The hybrid energy harvester combining two or more electromechanical conversion mechanisms has achieved different degrees of improvement in energy output, which is an effective scheme to improve energy harvesting efficiency. Wacharasindhu and Kwon [39] proposed a piezoelectric–electromagnetic hybrid energy harvester to harvest the energy generated by typing on the computer keyboard, and obtained maximum power of 40.8 μW and 1.15 μW from the piezoelectric module and the electromagnetic module, respectively. Later, Maharjan et al. [40] conducted research on energy harvesting during keyboard typing motion based on the principles of electromagnetic induction and triboelectric mechanism. Rajarathinam and Ali [41] studied a hybrid energy harvester that included a PZT cantilever beam and a magnet suspended by a spring. Sriramdas and Pratap [42] conducted experimental research on energy harvesting circuits with similar structures, and achieved an energy conversion efficiency of 50%. Although multiple energy conversion mechanisms can achieve efficient energy harvesting, current hybrid energy harvesters rarely involve multi-directional energy harvesting solutions.

In recent years, scholars have paid attention to self-adjusting or self-adaptive energy harvesting schemes, such as the omnidirectional piezoelectric energy harvester (PEH) with autonomous direction regulation capability proposed by Wang [43] and the directional self-adaptive piezoelectric energy harvester (DSPEH) proposed by Han [44]. Taking DSPEH as an example, this scheme adopts a two-degree-of-freedom (DOF) piezoelectric cantilever beam structure, which achieves multi-directional vibration energy harvesting under the action of centrifugal force and gravity by releasing the rotational constraint of the cantilever beam. However, the above directional self-adaptive energy harvesting scheme involves two processes, passive adjustment and steady-state vibration, and only the steady-state vibration process is the effective working state of the harvester. When the direction of motivation continues to change, the harvester will remain in an adjustment state, and even fail to adjust under certain extreme conditions. At present, the existing multi-directional or self-adaptive energy harvesting schemes all have active or passive perception and adaptation to external excitation directions. Here, this paper makes a bold conjecture: if a two-DOF cantilever beam is subjected to continuous or reciprocating rotational motion through some means, can the piezoelectric cantilever beam achieve both vibration and rotation, thereby achieving multi-directional and efficient energy harvesting?

That is, to achieve the opposite, the normal direction of the cantilever beam (bending vibration direction of the cantilever beam) must be maintained in a constantly changing state, and the influence of external excitation direction on the cantilever beam must be eliminated, rather than adapting to the external excitation direction. This article draws inspiration from the bi-stable structure in wideband energy harvesters and introduces end magnetic oscillators on the basis of DSPEH. Nonlinear magnetic forces cause the rotational DOF of the cantilever beam to be in an unstable state, resulting in rotational motion. The cantilever beam will produce a large amplitude response under the action of magnetic force, and the rotating cantilever beam eliminates the influence of excitation direction on the normal vibration of the cantilever beam, so that the cantilever beam can efficiently harvest vibration energy in the continuous direction. At the same time, the rotational energy of the cantilever beam in terms of rotational DOF can be harvested using electromagnetic or triboelectric mechanisms, which can further improve the energy harvesting efficiency of the system.

On the basis of directional self-adaptive energy harvesting, this paper proposes a multi-directional hybrid energy harvester (MHEH), which simultaneously uses the piezoelectric and electromagnetic mechanisms to harvest the energy of the vibrational and rotational DOFs from one excitation to improve the energy harvesting efficiency. This paper is organized as follows. Section 2 describes the design scheme and the working principle of the proposed harvester. Section 3 presents the experimental setup. Section 4 reveals the dynamic response of the hybrid energy harvester as well as the comparison between the proposed MHEH and the DSPEH. Section 5 draws conclusions.

## 2. Design Scheme and Working Principle

**Figure 1:**
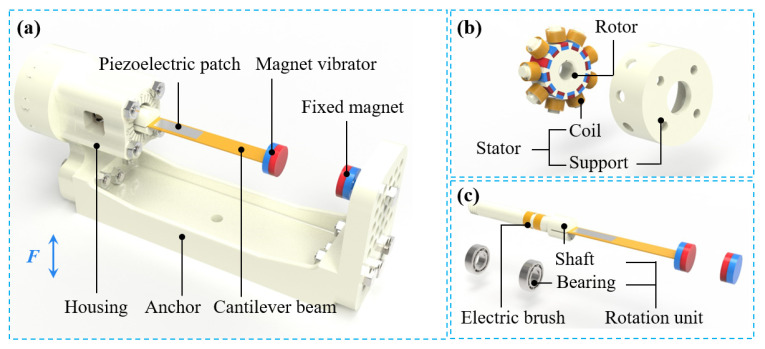
(**a**) Structure diagram of the MHEH; (**b**) detailed drawing of the electromagnetic module; (**c**) detailed drawing of the piezoelectric module.

The structure of the MHEH is shown in Figure 1, mainly composed of a piezoelectric module for harvesting the vibration energy of the cantilever beam (vibrational DOF) and an electromagnetic module for harvesting the rotational energy of the cantilever beam (rotational DOF).

The piezoelectric module is similar to the DSPEH [44], mainly including a cantilever beam, a piezoelectric patch, a magnet vibrator, a fixed magnet, a rotation unit, etc. A cylindrical magnetic vibrator is installed at the end of the cantilever beam. On the one hand, the magnetic vibrator acts as a tip mass, which is used to increase the amplitude of the cantilever beam and improve the piezoelectric output performance. On the other hand, it can generate variable nonlinear magnetic force under the action of fixed magnetic force, and enhance the instability of the system. The cantilever beam with the piezoelectric patch and the magnetic vibrator is fixed on the shaft of the rotation unit, and the shaft is installed on a housing through a pair of bearings. The housing and the anchor are mounted as a whole, and the fixed magnet is installed on the anchor. Based on the piezoelectric effect of the piezoelectric material, the piezoelectric module uses the bending deformation of the cantilever beam to drive the deformation of the piezoelectric material, thus generating electrical energy output, which is completely the same as the conventional piezoelectric energy harvester. Different from the DSPEH, because the cantilever beam will generate continuous rotational motion, an electric brush structure is designed on the rotation unit to facilitate the harvesting of the output electrical energy of the piezoelectric patch.

The electromagnetic module is mainly composed of a stator and a rotor: the stator is arranged with nine coil units, which are connected in series with each other, and each coil unit includes a coil bracket and a group of coils; 12 rectangular magnets with staggered polarity are installed on the rotor. The stator is fixedly connected with the anchor through a bracket, and the rotor is installed on the rotation unit of the piezoelectric module. The magnet array on the rotor rotates with the rotation unit, which changes the magnetic flux of the coil on the stator and generates the induced electromotive force, thus converting the rotational energy into electrical energy.

The working principle of MHEH can also be qualitatively explained using the theoretical formula of DSPEH. According to Figure 1 and references [44,45], the magnetic vibrator is installed at the center of the end of the cantilever beam, with a mass eccentricity coefficient of 0. Neglecting frictional torque and introducing nonlinear magnetic force, the dynamic equation can be described as:

(1) $$\left\{\begin{array}{l} m\ddot{x}+c_{1}\dot{x}+kx-mx{\dot{\theta }}^{2}+mg\left(1-\cos\theta \right)+\alpha V+F_{m}=F\cos\theta \\ mx^{2}\ddot{\theta }+c_{2}\dot{\theta }+2mx\dot{x}\dot{\theta }+mgx\sin\theta +\beta I+T_{m}=Fx\sin\theta \\ C_{P}\dot{V}-\alpha \dot{x}+\frac{V}{R_{1}}=0\\ L_{0}\dot{I}-\beta \dot{\theta }+R_{2}I=0 \end{array}\right.$$

where *m* and *k* are the equivalent mass and equivalent stiffness of the cantilever beam, respectively; *c*_1_ and *c*_2_ are the viscous damping coefficients in the vibrational DOF and rotational DOF of the cantilever beam, respectively; *V* represents the output voltage of the piezoelectric module; *I* represents the output current of the electromagnetic module; *R*_1_ and *R*_2_ represent the equivalent resistance of the piezoelectric and electromagnetic energy harvesting circuits, respectively; *α* and *β* represent the equivalent electromagnetic coefficient; *F* represents the intensity of external excitation; and *F_m_* represents the component of magnetic force on the vibrational DOF, and *T_m_* represents the component of magnetic moment on the rotational DOF, both of which are functions of *x* and *θ*.

Under ideal installation conditions, the rotational motion of the cantilever beam will not affect the component of the magnetic coupling effect on the rotational DOF, that is, *T_m_* = 0. According to the research of bi-stable system, the component of magnetic coupling effect on the vibrational DOF can be expressed as *F_m_* =*ax* + *bx*^3^, where *a* and *b* are constants, which are related to the magnet spacing. Equation (1) can be reformatted as the following non-dimensional forms.

(2) $$\left\{\begin{array}{l} \ddot{x}+\varsigma_{1}\dot{x}+\left(\omega_{10}^{2}+am^{-1}\right)x-x{\dot{\theta }}^{2}+\left(g+f\right)\left(1-\cos\theta \right)+\kappa_{1}y+bm^{-1}x^{3}=f\\ \ddot{\theta }+\varsigma_{2}\dot{\theta }x^{-2}+2\dot{x}\dot{\theta }x^{-1}+\left(g-f\right)x^{-1}\sin\theta +\kappa_{2}zx^{-2}=0\\ \dot{y}+\chi_{1}y-\dot{x}=0\\ \dot{z}+\chi_{2}z-\dot{\theta }=0 \end{array}\right.$$

where

$$y=\frac{C_{P}}{\alpha }V,z=\frac{L_{0}}{\beta }I,\varsigma_{1}=\frac{c_{1}}{m},\varsigma_{2}=\frac{c_{2}}{m},\omega_{10}^{2}=\frac{k}{m},f=\frac{F}{m},\kappa_{1}=\frac{\alpha^{2}}{mC_{p}},\chi_{1}=\frac{1}{R_{1}C_{P}},\kappa_{2}=\frac{\beta^{2}}{mL_{0}},\chi_{2}=\frac{R_{2}}{L_{0}}.$$

Expand the trigonometric function according to Taylor series, the equation is further simplified as:

(3) $$\left\{\begin{array}{l} \ddot{x}+\varsigma_{1}\dot{x}+\left(\omega_{10}^{2}+am^{-1}\right)x-x{\dot{\theta }}^{2}+\left(g+f\right)\left(\theta^{2}/2-\theta^{4}/24+\cdots \right)+\kappa_{1}y+bm^{-1}x^{3}=f\\ \ddot{\theta }+\varsigma_{2}\dot{\theta }x^{-2}+2\dot{x}\dot{\theta }x^{-1}+\left(g-f\right)x^{-1}\left(\theta -\theta^{3}/6+\cdots \right)+\kappa_{2}zx^{-2}=0\\ \dot{y}+\chi_{1}y-\dot{x}=0\\ \dot{z}+\chi_{2}z-\dot{\theta }=0 \end{array}\right.$$

**Figure 2:**
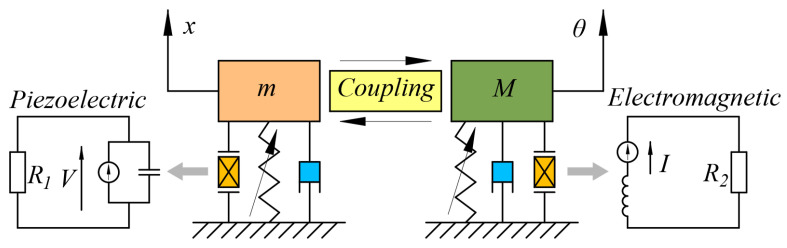
Simplified model of the proposed harvester.

It can be seen from Equation (3) that the left side of the equation is a function of *x* and *θ*; the right side of the equation is the driving force, and there is only driving force on the vibrational DOF. So, the energy harvesting system can be simplified to the model shown in Figure 2, where (*x*, *θ*) is the displacement in the generalized coordinate system, which represents the displacement in the vibrational DOF and rotational DOF, respectively; (*m*, *M*) is the generalized mass, which represents the equivalent mass and the equivalent moment of inertia of the system, respectively. The displacement in the vibrational DOF is directly driven by the external excitation *f*, which can produce continuous vibration; the displacement in the rotational DOF is driven by the *x* coupling term, and its vibration state depends on the coupling strength. According to Equation (3), the natural frequency of the vibrational DOF is related to the natural frequency of the cantilever beam *ω*_10_ and the magnetic force coefficient *a*. It can be seen that the magnetic force can adjust the natural frequency of the vibrational DOF, change the proximity to the external excitation, and then affect the stability of the system.

## 3. Prototype and Experimental Setup

**Figure 3:**
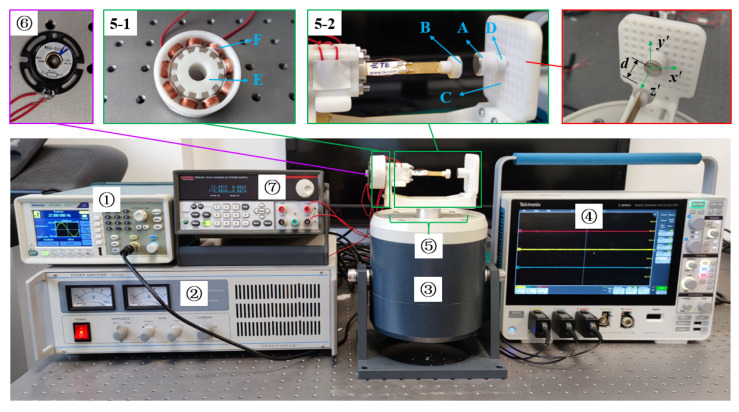
Experimental device diagram of the harvester. ① Signal generator. ② Power amplifier. ③ Electromagnetic exciter. ④ Oscilloscope. ⑤ MHEH: 5-1 electromagnetic module; 5-2 piezoelectric module (A: fixed magnet, B: magnet vibrator, C: support plate, D: shim, E: rotor, F: stator). ⑥ Angle sensor. ⑦ DC power supply.

The prototype of the MHEH is shown in Figure 3, which is mainly composed of piezoelectric module 5-1 and electromagnetic module 5-2, and its main parts include cantilever beam, magnet vibrator, fixed magnet, piezoelectric patch, rotor, stator, etc. Among them, the magnetic vibrator B at the end of the cantilever beam vibrates and rotates with the cantilever beam; the support plate C with fixed magnet A is fastened with the anchor, and the magnet spacing between magnet vibrator and fixed magnet in the z’ direction is adjusted by adjusting shim D. The stator F of the electromagnetic module is composed of nine coil units fixed on the coil frame in series, and the coil frame is installed on the anchor through bolts and nuts. The rotor E is composed of 12 rectangular magnets with magnetic staggered arrangement and is installed on the rotating unit through the stator frame. Considering the significant bending deformation of cantilever beams under nonlinear magnetic forces and the cost of use, PVDF is selected as the piezoelectric material in this paper. Because the MHEH scheme proposed in this paper is obtained by adding nonlinear magnetic force on the basis of DSPEH, the piezoelectric parameters, except for the length of the piezoelectric patch, are the same as those of DSPEH. Using information from reference [44], the relevant parameters of the MHEH are shown in Table 1 and Table 2.

The experimental device includes a signal generator, a power amplifier, an electromagnetic exciter, and an oscilloscope. The signal generator is used to send out a sine signal, and the power amplifier amplifies and transmits the sine signal to the electromagnetic exciter. The hybrid energy harvester is installed on the electromagnetic exciter and outputs the voltage signals of the piezoelectric module and the electromagnetic module to the oscilloscope. The rotation of the cantilever beam is monitored by the angle sensor and transmitted to the oscilloscope. The angle sensor belongs to a magnetic sensitive sensor, with a measurement angle range of 0–360°, a measurement accuracy of 0.05%, and a signal temperature drift of 60 μA. The sensor requires 24 V DC power supply, so the experimental device is equipped with a DC power supply.

## 4. Dynamic Response of the Hybrid Energy Harvester

**Figure 4:**
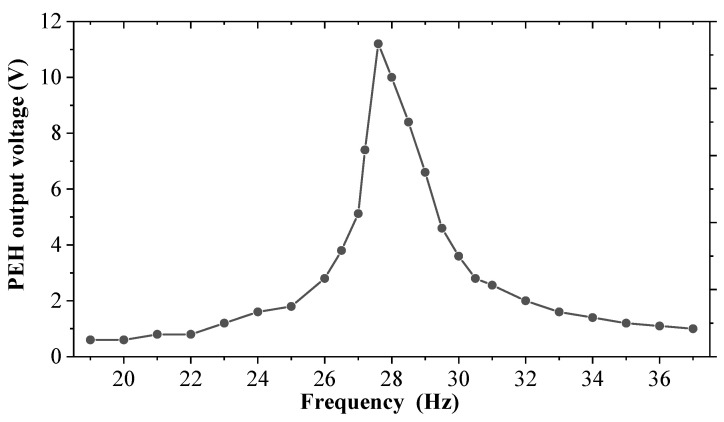
Frequency sweep curve of the conventional PEH.

The conventional PEH with the same parameters as the piezoelectric module is first swept experimentally to select an appropriate excitation frequency and facilitate comparison. As shown in Figure 4, during the excitation acceleration of 0.7 g, the resonance frequency of the conventional PEH is about 27.5 Hz, and the output voltage amplitude is about 11.3 V. In this paper, in order to verify the effect of magnetic force in the hybrid energy harvester, the excitation frequency is chosen as 27.3 Hz, under which the cantilever beam of the proposed harvester will not rotate if there is no magnetic force.

**Figure 5:**
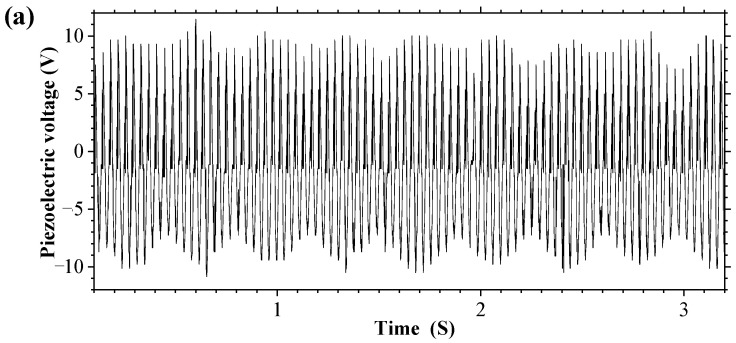

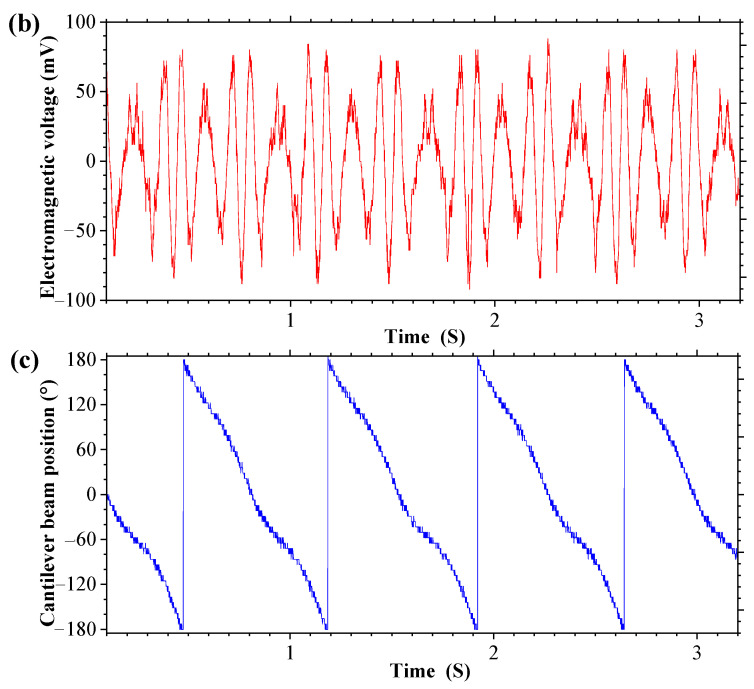
Output response of the hybrid energy harvester: (**a**) piezoelectric module voltage; (**b**) electromagnetic module voltage; (**c**) cantilever beam position.

When the excitation frequency is 27.3 Hz and the magnet spacing *d* = 24 mm (magnet spacing *d* is defined as the center distance between magnet vibrator B and fixed magnet A), the output response of the MHEH is shown in Figure 4, in which Figure 5a shows the change curve of the output voltage of the piezoelectric module with time, Figure 5b shows the change curve of the output voltage of the electromagnetic module with time, and Figure 5c shows the change curve of the cantilever beam position with time. Due to the use of an angle sensor with a range of 0–360° in the experiment, 180° and −180° indicate that the cantilever beam is in the same position. Therefore, it can be seen from Figure 5c that the cantilever beam has continuous rotational motion. The continuous rotational motion of the cantilever beam causes the voltage output peak value of the piezoelectric module to fluctuate, as shown in Figure 5a, which is different from the steady-state vibration of the DSPEH. However, due to the effect of magnetic force, the output of the piezoelectric module is still at a very high level. In addition, due to the rotational motion of the cantilever beam, the electromagnetic module also has continuous voltage output, and simultaneous energy harvesting from one excitation through two mechanisms is achieved.

### 4.1. Impedance Matching

**Figure 6:**
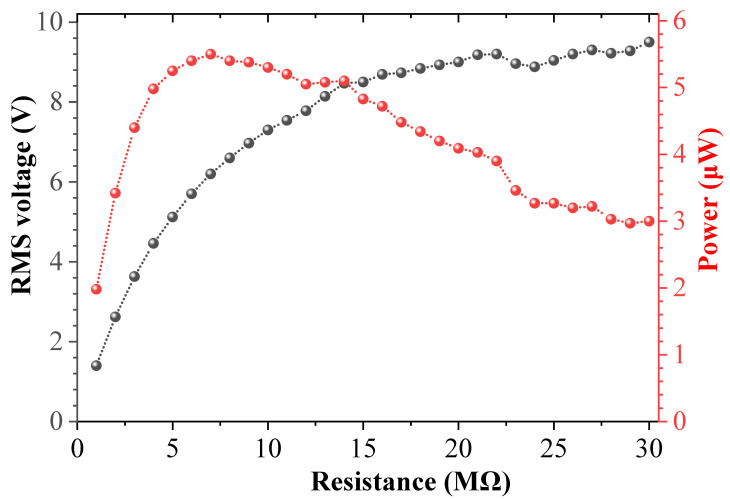
Output voltage and power of piezoelectric module under different resistance loads.

**Figure 7:**
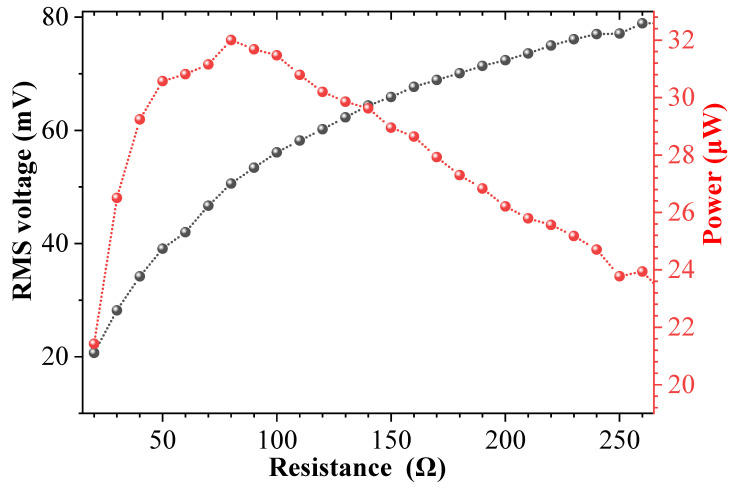
Output voltage and power of electromagnetic module under different resistance loads.

In order to obtain the optimal numerical output, the impedances of the piezoelectric module and the electromagnetic module are optimized before the experiment. Figure 6 and Figure 7 show the variation curve of the output voltage and average power of the piezoelectric module and the electromagnetic module with the resistance load under the same excitation frequency and acceleration, respectively. The excitation frequency selected in the experiment is 27.3 Hz, the excitation acceleration is 0.7 g, and the magnet spacing *d* is 24 mm. The experimental results show that the output voltage increases with the increase in the resistance load, and the output power first increases and then decreases with the increase in the resistance load. When the resistance load of the piezoelectric module is 7.0 MΩ, the maximum average power available is about 5.5 μW; when the resistance load of the electromagnetic module is 80 Ω, the maximum average power available is 32 μW. This phenomenon can be explained as follows: the output power of the system is related to voltage and resistance, and is proportional to the square of voltage and inversely proportional to resistance. Although the output voltage increases with the increase in the resistance load, the slope of the voltage change in the y-axis decreases, and the voltage curve becomes smoother, while the resistance change in the x-axis is linear. Therefore, the output power first increases and then decreases. Because the piezoelectric material used in the experiment is PVDF, its electromechanical coupling coefficient and the adhesion of the piezoelectric layer are poor, and compared with the length of the piezoelectric patch in DSPEH, the length of the piezoelectric patch used in this paper is only 10 mm, so the average power generated by the piezoelectric module of the prototype is not high in magnitude. If more efficient piezoelectric materials (such as MFC, etc.) are used, the output power of the piezoelectric module will be greatly improved [46].

### 4.2. Magnet Spacing Analysis

**Figure 8:**
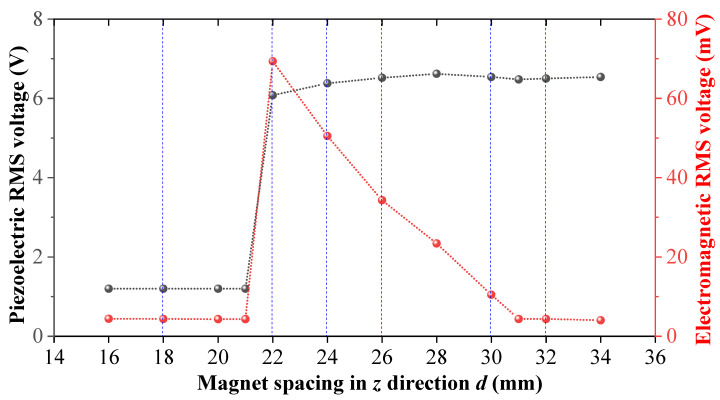
RMS voltage output by piezoelectric module and electromagnetic module under different magnet spacing.

**Figure 9:**
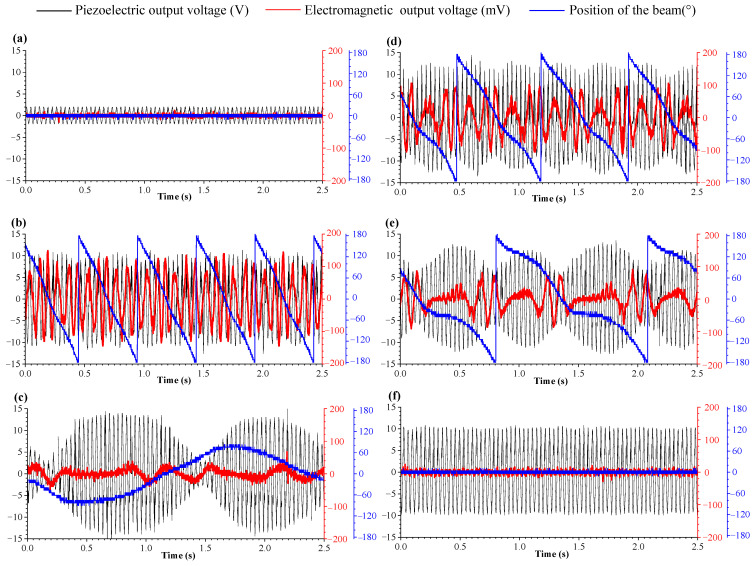
Output response of the system with different magnet spacing: (**a**) *d* = 18 mm; (**b**) *d* = 22 mm; (**c**) *d* = 24 mm; (**d**) *d* = 26 mm; (**e**) *d* = 30 mm; (**f**) *d* = 32 mm.

In a bi-stable system, the magnet spacing has a great influence on the energy output characteristics of the system, and proper magnet spacing can increase the amplitude response and increase the working bandwidth of the system. Similarly, the magnet spacing in the MHEH proposed in this paper also has a great impact on the output response of the system. Figure 8 and Figure 9 show the output response of the piezoelectric module and electromagnetic module under different magnet spacing.

It can be seen from Figure 9a that when the magnet spacing is less than 22 mm, due to the large potential energy between two magnets, the cantilever beam cannot break through the magnetic potential barrier, which leads to a small amplitude response of the cantilever beam and there is no rotational movement in the rotational DOF, so the output voltage amplitude of the piezoelectric module is very small and the electromagnetic module has no voltage output. When the magnet spacing reaches the limit of 22 mm, the cantilever beam breaks through the magnetic potential barrier and starts to vibrate with a large amplitude, similar to the bi-stable vibration system. Under the effect of magnetic force, the cantilever beam generates rotary motion with high frequency, and the output response of the electromagnetic module is considerable, as shown in Figure 9b. When the magnet spacing increases, the magnetic force between two magnets decreases, and the rotation frequency of the cantilever beam decreases, so the output electrical response of electromagnetic module decreases, as shown in Figure 9c,d. According to the research on DSPEH in reference [44], the position of the cantilever beam affects the voltage output amplitude, so it can be seen from the figure that the decrease in the rotating frequency of the cantilever beam makes the output voltage amplitude of the piezoelectric module fluctuate more and more obviously. When the magnet spacing is further increased, the effect of magnetic force can only drive the cantilever beam to swing back and forth, and cannot complete the rotational motion. The RMS output voltage of the electromagnetic module is only 10.46 mV, and the peak and valley of the piezoelectric module are more obvious, as shown in Figure 9e. When the magnet spacing is greater than 30 mm, the magnetic force has almost no effect on the vibration of the cantilever beam, which can only vibrate stably at a fixed position without rotating movement, and the hybrid energy harvester becomes a directional self-adaptive energy harvester, as shown in Figure 9f.

According to Figure 8 and Figure 9, the cantilever beam produces different rotation forms under different magnet spacing, and the output power of the electromagnetic module decreases rapidly with the distance increasing. Although the rotation form of the cantilever affects the output response of the piezoelectric module and makes its output voltage fluctuate, the peak voltage output also changes under the magnetic force, so the total energy output of the piezoelectric module is not different. As shown in Figure 9b,d, when *d* = 22 mm, the output voltage peak value of the piezoelectric module is 10.8 V, and when *d* = 26 mm, the output peak value of the piezoelectric module is 13 V; the RMS voltage of the two modules is 6.08 V and 6.52 V, respectively, with a difference of 6.7%.

### 4.3. Analysis and Evaluation of Energy Harvesting Performance

**Figure 10:**
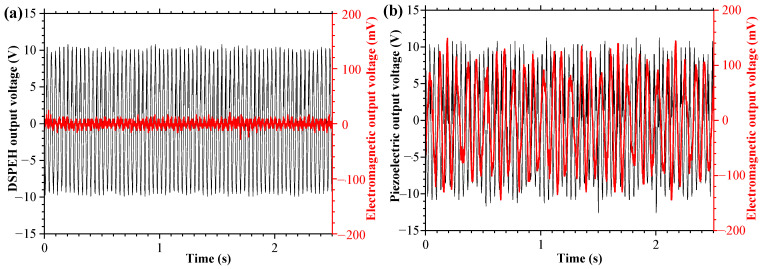
Comparison of energy harvesting output performance: (**a**) DSPEH; (**b**) MHEH.

According to the above analysis, under the condition of the magnet spacing *d* = 22 mm, the output performances of the MHEH and DSPEH with the same parameters as the piezoelectric module are compared in Figure 10. The RMS voltage and average power of the DSPEH are 6.55 V and 6.2 μW, respectively. After adding the electromagnetic energy harvesting device, the MHEH effectively utilizes the rotational energy in the rotational DOF. The RMS voltage of the piezoelectric module and the electromagnetic module are 6.08 V and 69.36 mV, respectively, and the average power is 5.3 μW and 61 μW. Although the piezoelectric module is slightly lower than the DSPEH, the total power of the system is increased by about 10 times. Considering the complex structure and large size of the MHEH, the power density of the two is 61.2 mW/m^3^ and 361.1 mW/m^3^, and the energy harvesting efficiency of the MHEH has been improved by about 500% from the perspective of power density.

**Figure 11:**
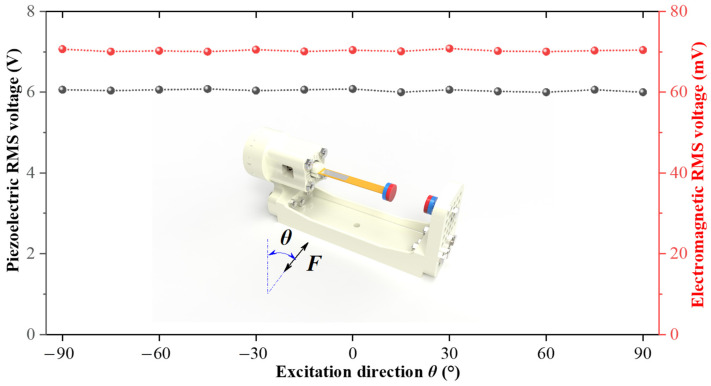
Output response of the system under different excitation directions.

When the MHEH works, the two-DOF cantilever beam has continuous vibration, so the output response of the system has little relationship with the direction of external excitation. Figure 11 shows the RMS output voltage curves of the piezoelectric module and electromagnetic module under different excitation directions. It can be seen from the figure that the MHEH still has the function of multi-directional energy harvesting due to the continuous rotational motion on the rotational DOF, and has similar high-power output in any excitation direction in two-dimensional space, which are the same characteristics as the DSPEH.

In summary, compared with the DSPEH we proposed earlier, the harvesting efficiency of the MHEH has been improved by 500% and the harvester has the same functions of multi-directional energy harvesting and self-adaptive energy harvesting as the previous scheme. If the stator of the electromagnetic module is integrated into the housing and the rotor is used as the rotating unit, the size of the prototype will be greatly reduced, which can achieve higher power density and further improve the energy harvesting efficiency.

## 5. Conclusions

Based on the research on DSPEH, this paper put forward a structural design scheme of hybrid energy harvesting, which simultaneously harvests the vibration energy in both directions of vibrational DOF and rotational DOF. The experimental study found that the continuous rotational motion of the two-DOF cantilever beam can be realized by adding the nonlinear magnetic force, and then the energy harvesting on the rotational DOF of the cantilever beam can be carried out. The addition of nonlinear magnetic force can adjust the natural frequency of the cantilever beam, change the stable state of the system, and then affect the energy output characteristics of the rotational DOF. Different magnet spacing corresponds to different rotational forms of the cantilever beam in the rotational DOF: when the cantilever beam breaks through the magnetic potential energy, the output power of the electromagnetic energy harvesting module decreases with the increase in the magnet spacing; when the magnet spacing increases to a certain value, the magnetic force will not affect the cantilever beam, and the cantilever beam can only vibrate stably at a fixed position. Compared with the DSPEH, after adding the electromagnetic module, the MHEH harvester effectively uses the rotational energy in the rotational DOF, and the output power of the electromagnetic module reaches 61 μW. The total power of the system is increased by 10 times, the power density is increased by about 500%, and the system has the function of multi-directional and self-adaptive energy harvesting.

However, the vibration and rotation of the cantilever beam are mutually influenced, not only by the changes in voltage amplitude as shown in Figure 9, but also by the coupling effects of piezoelectric and electromagnetic circuits. Effectively utilizing the coupling effects between the two can effectively improve energy harvesting efficiency, which will be further studied in our next work. In addition, the rotational energy of the cantilever beam is harvested through electromagnetic mechanism. In practical applications, mechanisms such as triboelectric, electrostatic, or a combination of multiple mechanisms can be selected to further improve energy harvesting efficiency. The research in this paper provides a new idea for multi-directional energy harvesting and realizes the functions of multi-directional and hybrid energy harvesting of the conventional cantilever beam. The scheme proposed in this paper applies a simple single cantilever beam structure, and its multi-directional and hybrid energy harvesting methods can be extended to series, parallel, nonlinear, and other energy harvesters, which will play an important role in the energy supply module of wireless sensor networks and Internet of Things systems.

## Figures and Tables

**Table 1:** Parametric values of the piezoelectric module.

**Cantilever Beam**		**Piezoelectric Patch**	
Parameters	Values	Parameters	Values
Material	Brass	Material	PVDF
Density (10^3^ kg/m^3^)	8.6	Length *L_p_* (m)	0.010
Length *L* (m)	0.052	Width *W_p_* (m)	0.008
Width *W* (m)	0.008	Thickness *H_p_* (m)	0.000028
Thickness *H* (m)	0.0006	Equivalent electromechanical coupling coefficient *α* (10^−5^ cm^−1^)	−7.333
Elastic modulus *E* (GPa)	120	Equivalent capacitance *C_P_* (10^−9^ F)	6.25
Damping coefficient *c*_1_ (Ns/m)	0.1	Equivalent resistance of the energy harvesting circuit *R* (10^6^ Ω)	7.0
**Magnet Vibrator**		**Fixed Magnet**	
Parameters	Values	Parameters	Values
Material	NdFeB	Material	NdFeB
Density (10^3^ kg/m^3^)	7.5	Density (10^3^ kg/m^3^)	7.5
Diameter (m)	0.012	Diameter (m)	0.012
Height (m)	0.005	Height (m)	0.005
Magnetization (10^5^ A/m)	2.12	Magnetization (10^5^ A/m)	2.12

**Table 2:** Parametric values of the electromagnetic module.

Rotor Magnet		Stator Coil	
Parameters	Values	Parameters	Values
Material	NdFeB	Material	Brass + polyurethane
Density (10^3^ kg/m^3^)	7.5	Coil diameter (m)	0.0001
Length × width × height (m × m × m)	0.01 × 0.005 × 0.003	Coil length (m)	5.65
Magnetization (10^5^ A/m)	1.21	Coil turns	150

## Data Availability

Data is contained within the article.

## Abbreviations

The following abbreviations are used in this manuscript:

DOF	Degree of freedom
DSPEH	Directional self-adaptive piezoelectric energy harvester
MHEH	Multi-directional hybrid energy harvester
PEH	Piezoelectric energy harvester
